# Rapid Diagnosis of Drug-Resistant Tuberculosis–Opportunities and Challenges

**DOI:** 10.3390/pathogens13010027

**Published:** 2023-12-27

**Authors:** Kogieleum Naidoo, Rubeshan Perumal, Senamile L. Ngema, Letitia Shunmugam, Anou M. Somboro

**Affiliations:** 1 Centre for the AIDS Programme of Research in South Africa (CAPRISA), Nelson R Mandela School of Medicine, University of KwaZulu-Natal, Durban 4001, South Africasenamile.ngema@caprisa.org (S.L.N.); letitia.shunmugam@caprisa.org (L.S.); anou.somboro@caprisa.org (A.M.S.); 2 SAMRC-CAPRISA HIV-TB Pathogenesis and Treatment Research Unit, Doris Duke Medical Research Institute, University of KwaZulu-Natal, Durban 4001, South Africa

**Keywords:** diagnosis, multidrug-resistant tuberculosis (MDR-TB), drug susceptibility test (DST), tuberculosis treatment, phenotypic resistance, genotypic resistance

## Abstract

Global tuberculosis (TB) eradication is undermined by increasing prevalence of emerging resistance to available drugs, fuelling ongoing demand for more complex diagnostic and treatment strategies. Early detection of TB drug resistance coupled with therapeutic decision making guided by rapid characterisation of pre-treatment and treatment emergent resistance remains the most effective strategy for averting Drug-Resistant TB (DR-TB) transmission, reducing DR-TB associated mortality, and improving patient outcomes. Solid- and liquid-based mycobacterial culture methods remain the gold standard for *Mycobacterium tuberculosis* (MTB) detection and drug susceptibility testing (DST). Unfortunately, delays to result return, and associated technical challenges from requirements for specialised resource and capacity, have limited DST use and availability in many high TB burden resource-limited countries. There is increasing availability of a variety of rapid nucleic acid-based diagnostic assays with adequate sensitivity and specificity to detect gene mutations associated with resistance to one or more drugs. While a few of these assays produce comprehensive calls for resistance to several first- and second-line drugs, there is still no endorsed genotypic drug susceptibility test assay for bedaquiline, pretomanid, and delamanid. The global implementation of regimens comprising these novel drugs in the absence of rapid phenotypic drug resistance profiling has generated a new set of diagnostic challenges and heralded a return to culture-based phenotypic DST. In this review, we describe the available tools for rapid diagnosis of drug-resistant tuberculosis and discuss the associated opportunities and challenges.

## 1. Introduction

Global TB control efforts are undermined by the increasing global prevalence of emerging resistance to available TB drugs [1]. Drug-resistant TB (DR-TB) is associated with poorer clinical outcomes than drug-susceptible TB and has created an ongoing demand for more complex diagnostic and treatment strategies. In 2021, 10.6 million people fell ill with TB [2], with three million amongst these either missed or undiagnosed. Among the estimated 450,000 cases of multidrug-resistant/rifampicin-resistant TB (MDR-TB/RR), only 166,991 (37.1%) people were diagnosed and referred onward for treatment, while MDR/RR-TB-related deaths were estimated at 191,000 people (42.4%) in the same year [2]. Thus, improvements in DR-TB diagnosis with rapid characterisation of drug resistance profiles are urgently required to enable the prompt initiation of therapy, reduce DR-TB transmission, and improve clinical outcomes.

Tuberculosis treatment has undergone significant changes in recent years, made possible by the discovery of novel anti-tuberculous drugs and driven by the expansion of drug resistance [3]. Furthermore, DR-TB guidelines have undergone several revisions recently to reflect the shifts in antimicrobial resistance evolution, the global transition away from toxic injectable drugs, and the improved availability of novel drug regimens. In the most recent iteration, multidrug-resistant TB (MDR-TB) is defined by the presence of resistance to at least isoniazid (INH) and rifampicin (RIF). Pre-extensively drug-resistant TB (pre-XDR-TB) is defined by resistance to RIF and at least one fluoroquinolone (FQ), while extensively drug-resistant TB (XDR-TB) is defined by resistance to RIF, at least one FQ, and another group A drug (that is, bedaquiline (BDQ) or linezolid (LZD)). Furthermore, RIF monoresistant TB and INH monoresistant TB represent distinct clinical entities for which optimal therapeutic strategies are yet to be defined [4,5].

Various factors are associated with the emergence and subsequent transmission of DR-TB, including adherence challenges, suboptimal pharmacotherapy from incorrect prescribing, pharmacokinetic–pharmacodynamic mismatch, and failure of timely DR-TB diagnosis before secondary cases are spawned [6,7]. The emergence and propagation of DR-TB have a significant economic and social impact. Firstly, managing DR-TB is expensive and varies according to the extent of resistance; direct treatment costs per case of MDR and extensively drug-resistant TB (XDR-TB) were estimated at USD 182,000 and USD 568,000 in the US, respectively [8]. Moreover, for a curable infectious disease, the duration of treatment is relatively long and includes drugs with significant toxicity, contributing to loss of productivity and income and an increased risk of experiencing adverse events [8]. Presently, the most effective strategy for mitigating the spread of DR-TB requires early detection of DR-TB cases, rapid characterisation of resistance profiles, prompt initiation of a safe and effective treatment regimen, close monitoring of treatment response, and the implementation of DR-TB preventative therapy in eligible contacts of index cases.

Given the centrality of early diagnosis of DR-TB and access to a clinically actionable profile of drug resistance, we reviewed the available diagnostic tools for DR-TB and provide a discussion on the opportunities and challenges for global programmatic implementation associated with each assay.

## 2. Search Strategy

We performed a search of published studies on PubMed, Google Scholar, and Web of Science using the following keywords: (tuberculosis) AND (resistan*) AND ((diagnos*) OR (molecular) OR (point of care)). The search was limited to articles published in or translated to English from inception until July 2023. We manually searched all references of included articles to identify additional articles not captured by the electronic search. We included all manuscripts reporting on at least one diagnostic test for the detection of MTB and resistance to at least one TB drug.

## 3. Principles of Drug Susceptibility Testing

In line with the World Health Organisation (WHO) recommendations, rapid TB diagnosis and universal drug susceptibility testing (DST) should be achieved systematically for anyone presenting with clinical symptoms of TB [9]. Resistance profiling requires bacteriological confirmation of TB followed by the detection of drug resistance using phenotypic (culture or phage-based) methods that observe mycobacterial growth in drug-containing media or genotypic (molecular or sequencing-based) methods that detect genes known to confer drug resistance [10]. Drug susceptibility testing for available anti-TB drugs is of critical importance; firstly, it helps guide the choice of chemotherapy; secondly, it is useful for confirming the emergence of drug resistance, especially in patients who are failing treatment; and thirdly, it can be used for surveillance of DR-TB at a programmatic level. Unfortunately, not all DST tools are readily available for clinical use as many high TB burden countries have limited resources and capacity for testing. Therefore, judicious selection of the diagnostic strategies for DR-TB must include consideration of local disease epidemiology, the relevance of diagnostic information for clinical decision making, the therapeutic armamentarium available for prescription, as well as an assay’s feasibility, cost, accuracy, and turnaround time within a given health system.

## 4. Phenotypic Drug Susceptibility Testing (pDST) Assays

In low- and middle-income countries (LMICs), acid-fast bacillus (AFB) microscopy remains the cornerstone of TB diagnosis. However, this method is limited by low sensitivity (60%), requires 5000 to 10,000 bacilli per mL of specimen to detect MTB on stained smears, and does not allow for DR-TB detection [11,12]. Pure mycobacterial cultures, including solid- and liquid-based methods, remain the gold standard for MTB detection and drug susceptibility testing (DST) (Table 1).

Phenotypic DST uses established critical concentrations (CCs), defined as the lowest concentration of the drug that will inhibit the growth of 99% of the wild-type population, to describe the phenotype of a particular strain [13]. In addition, the WHO has defined clinical breakpoints (CB) for some drugs (i.e., the concentration above the CC that separates susceptible strains from resistant strains), which may be used for clinical decision making. Growth above the CB indicates that the organism is less likely to respond to the drug at a given dose and, in some instances, that the drug may be used at a higher-than-usual dose to achieve efficacy [13]. In 2018, the WHO published a manual on established and interim CCs for DST for all drugs in groups A, B, and C [13,14]. Isoniazid (INH) and moxifloxacin (MFX) have CBs set for high- and low-level resistance, where concentrations of 0.1 mg/L and 0.4 mg/L in MGIT correspond to low-level resistance and high-level resistance for INH, respectively, and 0.25 mg/L and 1.0 mg/L correspond to low- and high-level resistance for MFX, respectively [14]. Drug resistance testing at a single CC or CB breakpoint may not be sufficient in guiding treatment, and while the MICs may provide a comprehensive resistance profile, this method is not part of the routine practise.

While critical concentrations of TB drugs have been established, the relevance of these concentrations to microbiologic and clinical outcomes is unclear since TB drugs are required to achieve a certain threshold exposure above the MIC at the site of infection to kill MTB effectively. Given that TB patients’ genetic background and anthropometry vary worldwide, pharmacokinetic variability that influences the achievement rate of concentrations above the MIC is considerable. Given that it is impractical to perform dose-ranging studies to establish the critical concentrations of tuberculosis drugs in individual patients, Monte Carlo simulations have been used to estimate the ability to achieve the antibiotic area under the concentration–time curve/MIC ratio associated with ≥90% of maximal kill (EC90) of MTB in ≥90% in the epithelial lining fluid of 10,000 tuberculosis patients, thereby establishing critical concentrations for the simulated population [15]. Failure to achieve EC90 in ≥90% of patients at a particular MIC was defined as drug resistance. This study found that the previously established critical concentrations of isoniazid of either 0.2 or 1.0 mg/L were far higher than the newly identified breakpoints of 0.0312 mg/L and 0.125 mg/L, defining low- and high-level isoniazid resistance, respectively. Based on these findings, the rates of multidrug-resistant tuberculosis are likely fourfold higher than previously anticipated.

Culture is not used as a primary diagnostic in many settings where the burden of TB is high due to high costs, burdensome infrastructure requirements, and the inherent delay in obtaining clinically actionable results (two to three weeks for positive results and up to six weeks for negative results). DST can only be performed once a positive culture is identified, and the results may take up to six weeks for solid media and 24 days for MGIT [13]. Culture contamination is a further threat to culture-based techniques. Culture DST, therefore, needs to be performed in a sterile area under approved and stringent laboratory conditions to minimise the risk of contamination. Nevertheless, without alternative tools, microscopy and culture methods remain necessary to monitor a patient’s response to treatment.

Solid Media: Two main types of solid media, egg-based (e.g., Lowenstein–Jensen) and agar-based (e.g., Middlebrook 7H10, 7H11), are endorsed by WHO for isolating MTB and determining susceptibility to anti-TB drugs [16]. Solid-based culture methods used to determine DR-TB include the proportion, absolute concentration, and resistance ratio methods [17,18]. Originally proposed by Canetti et al., the proportion method is performed by inoculating Lowenstein–Jensen (LJ) media, containing the CC of selected anti-TB drugs (test media) and drug-free media (control media), with culture suspension of MTB [19]. The growth of MTB in the drug-containing medium is then compared to the growth in the drug-free control medium. The ratio of the number of colonies on the drug-containing medium to the number of colonies on drug-free medium is calculated, and a 1% proportion threshold is used to define resistance (i.e., if 1% or more of the populations of MTB are resistant, the culture is reported as resistant, and conversely, if the ratio of the number of colonies on the medium containing an anti-TB drug to the number of colonies on the control medium is less than 1%, then the strain is reported as susceptible). The assay results can be provisionally reported after three weeks if there is growth on the drug-containing medium, while definitive results can only be reported after six weeks of incubation [20]. The proportion method is validated for most first- and second-line TB drugs using direct (e.g., sputum) or indirect (MTB isolates from culture) specimens [21,22]. To reduce the time to DR-TB detection, Amini et al., compared the performance of this method on direct clinical specimens (e.g., smear-positive sputum samples) versus culture isolates, and found complete concordance (100% specificity and sensitivity) between the two types of specimens with a mean detection time of 20 days [23]. The WHO, however, recommends the use of culture suspension rather than a direct clinical specimen for the performance of DST. However, for susceptibility to certain anti-TB drugs such as pyrazinamide (PZA), using the proportion methods is not ideal, as PZA requires low pH conditions that are not possible with media. Furthermore, newer anti-TB drugs such as bedaquiline, linezolid, pretomanid, delamanid, and clofazimine have not been validated for proportion DST testing using LJ media [13]. In agar-based 7H10, the CCs are well established for FQs and linezolid, and are set at 1.0, 0.5, and 1.0 mg/L for levofloxacin, moxifloxacin, and linezolid, respectively. The interim CC for delamanid in agar-based 7H11 is set at 0.016 mg/L, but has not yet been established for pretomanid.

The absolute concentration method uses a standardised inoculum of MTB isolate on media of varying drug concentrations and expresses resistance in terms of the lowest drug concentration that inhibits MTB growth (less than 20 colonies), reported as the minimum inhibitory concentration (MIC). The absolute concentration method is also commonly used based on its technical simplicity for inoculum preparation and result interpretation. Results are reported as susceptible (growth less than 20 colonies), total resistance (growth comparable to the control), or partial resistance (growth markedly less than the control) [17,19]. When resistance is reported at a MIC above the set CC or CB, the drug should not be used as part of a regimen without expert consultation. However, in cases where resistance is reported above CC but below the CB, a higher dose of the drug may be used to overcome resistance. Clinically permissible high-dose strategies are well established for moxifloxacin and INH [24]. This method is greatly affected by inoculum size and the organisms’ viability [17,18].

The resistance ratio method is a refinement of the absolute concentration method, in which variations in MIC for a given isolate are monitored when the isolate is tested on different concentrations of drug-containing media [25]. Resistance is expressed as the ratio of the test MTB strain’s MIC divided by the MIC of a reference MTB strain (e.g., H37Rv strain) [26]. The results are reported as fully susceptible if the ratio is two or less and highly resistant if the ratio is eight or more. Intermediate or low-level resistance is difficult to measure accurately with this method [18].

These solid-based culture pDSTs are labour intensive, require sophisticated laboratory infrastructure, and require visualisation of MTB growth, resulting in turnaround times of 3 to 6 weeks. The estimated cost per test for solid culture to isolate MTB and perform DST is ±22 USD [27]. As a result, the methods are rarely available outside centralised reference laboratories.

Liquid media: Suitable liquid media for mycobacteria growth include Middlebrook 7H9 broth, which is used for automated liquid culture methods (e.g., BACTEC MGIT, VersaTREK, MB/BacT ALERT) and the manual microdilution method (Sensititre). There is a notably lower rate of mycobacterial isolation and a longer time to detect DR-TB using solid media compared to liquid media [11,28]. The limit of detection (LoD) for liquid culture is down to 10 viable bacilli per millilitre (mL), while 100 bacilli per mL are necessary for solid culture [29]. Despite the availability of many different culture-based methods, liquid-based culture remains the most frequently used method in the world [30]. This is due to several reasons: (i) the system is highly automated and only requires a sample to be prepared and loaded into the instrument, (ii) faster growth of MTB, with the ability to provide DST results in 4–24 days in MGIT 960 [31], (iii) higher recovery rate of MTB strains compared to solid media [32], and (iv) very low LoD as the system uses fluorescence to monitor MTB growth.

BACTEC MGIT is a widely used and fully automated mycobacterial detection system for mycobacterial liquid culture and DST. The system offers mycobacterial susceptibility testing for first- and second-line anti-TB drugs, including PZA, FQs, bedaquiline, and linezolid [33,34,35]. The CC for pretomanid has not yet been established, while the interim CC for delamanid is 0.06 mg/L. The time to detection varies from around ten days for smear-positive samples to two–six weeks for smear-negative samples [36]. The average turnaround time for culture positivity and DST using liquid media is about 23 days [31], while for a solid (Lowenstein–Jensen) medium it is much longer (above 30 days). The WHO has recommended the BACTEC MGIT system for phenotypic DST and supports its implementation in peripheral laboratories. Drug susceptibility testing using direct patient specimens with this automated system offers further time savings and rapid, reliable detection of DR-TB. However, liquid culture methods are more prone to contamination, more costly (±29 USD per test for liquid culture plus DST) [27], and require dedicated laboratory training. The results are qualitative for liquid culture (positive/negative for MTB detection) and quantitative for solid cultures (number of colonies) [23,37].

Although considered the reference standard method for MTB isolation and detection of resistance to many anti-TB drugs, neither solid- nor liquid-culture-based pDST methods can produce clinically actionable results in near real time. Therefore, alternative methods for rapid TB diagnosis and accurate resistance profiling are required to support TB control efforts.

## 5. The Microscopic Observation Drug Susceptibility Assay (MODS)

The MODS assay is a liquid-culture-based test that detects MTB and assesses anti-TB drug susceptibility directly from sputum samples or from MTB isolates. The method uses two important properties of MTB: the faster growth in liquid media than solid media and the easily recognisable and characteristic microscopic cording appearance of that growth in liquid media. Commercially available 24-well microtiter plates containing supplemented Middlebrook 7H9 medium are inoculated with specimens and controls in corresponding drug-free and drug-containing wells and examined for microcolonies using an inverted light microscope. The microcolonies are detected in an average of seven days for smear-positive specimens, much earlier than macroscopic colony growth can be seen on solid media [38]. The concordance between MODS culture and the standard culture methods is around 97% for RIF, 95% for INH, and 98% for both RIF/INH [39,40]. MODS is a recommended assay by the WHO for directly testing sputum specimens. The assay is sensitive and validated for first- and second-line anti-TB drug susceptibility tests and could significantly improve DR-TB diagnosis in resource-limited settings. MTB isolation and DST are concurrent in MODS assay, using direct specimens, and the average time to DST results is the same as for detecting MTB with conventional liquid culture methods.

The simplicity of the technique, the greater sensitivity of liquid over solid medium culture for MTB detection, the specificity of the characteristic growth of MTB, and the evaluation of first- and second-line anti-TB drug susceptibility in a short timescale are the major advantages of the method. However, MODS requires additional skills in using inverted microscopy and additional consumables (microtiter plates) that may be difficult to obtain in some settings. It requires daily examinations after day five of incubation to monitor the assay, which may be time consuming and labour intensive. The biosafety risk associated with using the MODS platform is similar to that of conventional culture on solid media and, therefore, requires biosafety level two precautions. MODS is suitable for use at the reference laboratory level, and decentralisation to lower-level laboratories is not presently recommended.

## 6. The Nitrate Reductase Assay (NRA)

NRA is based on the ability of MTB to reduce nitrate to nitrite, which is used for the biochemical identification of mycobacterial species [41]. The diazotisation reaction, which constitutes the basis of the Griess test for nitrite detection, was first described by Griess in 1879 [42] and then subsequently evaluated as an MTB DST assay. The test is conducted by inoculating Lowenstein–Jensen medium containing a standardised concentration of potassium nitrate (KNO_3_), in the presence of anti-TB drugs at their critical concentrations. The nitrate reduction is then detected using Griess’ reagent, which produces a coloured reaction in which the appearance of a pinkish-red colour indicates drug resistance. Susceptible MTB strains, progressively killed or inhibited by the anti-TB drugs, no longer reduce nitrate and, therefore, fail to generate a colour change [43,44]. The NRA can be used directly on smear-positive sputum specimens or indirectly on MTB isolates grown from conventional solid culture. Therefore, DR-TB testing with NRA is not faster than the conventional phenotypic DST method unless performed on smear-positive sputum samples. Studies on direct and indirect tests showed that the pooled sensitivities were 96% for INH, 97% for RIF, 90% for ethambutol (EMB), and 82% for streptomycin (STR), and the pooled specificities were 99% for INH %, 100% for RIF, 98% for EMB, and 96% STR. The time to reportable results varied from 5 to 28 days for direct NRA and 5 to 14 days for indirect testing [41,45,46]. Furthermore, the NRA performance was also evaluated using a liquid medium to reduce the time to results. The finding showed that the liquid-based NRA had an accuracy of 97–100% for RIF, 96.8–99.2% for INH, 98–100% for ofloxacin (OFL), 96.8–98.5% for amikacin (AMK), 96.4–99.5% for kanamycin (KAN), and 96.8–100% for capreomycin (CAP), compared to the agar proportion method or MGIT 960 system. The median turnaround time for liquid-based NRA was seven days [47]. NRA performed in a liquid medium offers a rapid, economical, and feasible method for phenotypic DST in resource-limited settings. However, the method has not yet been validated for novel anti-TB drugs.

## 7. The Resazurin Microtiter Assay (REMA)

The REMA, initially used for measuring the viability of mammalian cells and bacteria, has recently been validated for DR-TB. Metabolically active cells reduce blue resazurin (nonfluorescent) to the pink product resorufin (fluorescent). This reduction is proportional to the number of metabolically active cells [48]. Therefore, MTB growth is monitored with resazurin reduction in the presence of anti-TB drugs to assess their susceptibility. Recent studies demonstrated that it is a reliable method for detecting DR-TB and showed a high level of agreement with nitrate reductase assays and the gold standard culture methods [48,49,50,51]. The assay results are easy to read by visualising a colour change of the reduced resazurin to resorufin. Alternatively, MTB growth can also be measured with a fluorometer or spectrophotometer. First- and second-line drug susceptibility can be assessed using REMA [49]. However, it remains to be validated for EMB, STR, and the new and repurposed anti-TB drugs.

A study evaluated the non-commercial pDST methods, including the microscopic observation drug susceptibility assay (MODS), nitrate reductase assay (NRA), and resazurin microtiter assay (REMA) using the conventional proportion method as a reference test for phenotypic DST in MTB clinical isolates. NRA was the most sensitive and specific among the evaluated assays, with a detection sensitivity of 100% for RIF resistance detection by NRA, 94.55% by REMA, and 92.73% by MODS. The specificity was 100% for NRA and REMA, while MODS was 98% specific for detecting RIF resistance. The time to results for all assays was 8–14 days [52]. All three assays are recommended by the WHO for rapid DST, are relatively inexpensive, and are suitable for use in LMICs.

## 8. Phage Assays

Phage assays are mycobacteriophage-based methods for rapid MTB detection and drug susceptibility testing [53]. A mycobacteriophage is a virus that specifically infects mycobacterial species and is usually engineered to carry a gene cassette encoding a sensitive reporter protein or enzyme [54]. The reporter protein is expressed in the presence of metabolic active mycobacteria, and the light produced can be measured with a luminometer. Luciferase-reporter mycobacteriophages (LRP) were reported three decades ago and are the most common [54]. Early studies on LRP demonstrated that MTB could be detected from freshly processed sputum, provided the bacillary load was >10^7^ [55]. Furthermore, incubating the sputum in a growth medium for a week improved the detection rate. Based on these results, phages have been optimised to reduce the incubation interval and improve their sensitivity [56]. Previously, a reporter phage was compared to GeneXpert MTB/RIF for the detection of MTB and RIF resistance. In smear-positive patients, the sensitivity of the reporter phage was 95.90%, and in smear-negative patients, it was 88.89% [57]. Low bacterial load has been reported as a challenge with this assay, with failure to detect mycobacteria when colony-forming unit counts are less than 10,000 CFU/mL at day zero [58]. Commercial kits based on phage technology, such as FASTPlaqueTB-RIF, PhageTek MB, and Actiphage, can detect mycobacteria in clinical samples [59]. Phage assays do not need to be performed by skilled personnel and do not require specialised equipment or expensive reagents. Furthermore, phage assays can produce DST results within 48 h when applied to culture isolates and are being optimised further to improve their performance on direct patient specimens [53,57,60]

## 9. Genotypic Drug Susceptibility Testing (gDST) Assays

The World Health Organisation (WHO) recently emphasised the need for countries to implement rapid molecular drug susceptibility tests (DSTs) for anti-TB drugs (e.g., RIF, INH, FQs). There is a rising number of rapid molecular diagnostic assays with adequate sensitivity and specificity for detecting MTB and profiling drug resistance (Table 1). Most of the recommended rapid molecular assays detect *Mycobacterium tuberculosis* complex (MTBc). Some assays additionally detect resistance to RIF, and some detect resistance to both RIF and INH, allowing mono- and multi-drug resistant TB detection. Moreover, some assays produce more comprehensive calls for resistance to several first- and second-line drugs [9]. However, gDST assays for the newly introduced molecules such as bedaquiline, pretomanid, and linezolid are challenging, predominantly because of insufficient data on their genotypic-phenotypic concordance for resistance. As a result, no rapid gDST assays have been endorsed for these drugs.

## 10. Probe-Based Assays

### 10.1. GenoType MTBDRplus and GenoType MTBDRsl (Bruker/Hain Lifescience, Nehren, Germany)

Genotype MTBDR assays are line probe assays (LPAs), that include GenType MTBDR*plus* for detecting INH and RIF resistance by identifying mutations in the *katG*, *inhA*, and *rpoB* genes, and GenoType MTBDR*sl* for detecting resistance to FQs, ethambutol (EMB), aminoglycosides (kanamycin, amikacin; viomycin), and cyclic peptide (capreomycin) by identifying mutations in three different loci, that are *gyrA*, *embB*, and *rrs*, respectively [61].

GenoType MTBDR*plus* (RIF, INH) is a first-line LPA that enables rapid diagnosis of TB from both direct pulmonary patient samples and culture isolates of MTB and detects resistance to RIF and INH. The identification of RIF resistance is enabled by detecting the most significant mutations of the *rpoB* gene (coding for the β-subunit of the RNA polymerase). For detecting high-level INH resistance, the *katG* gene (coding for the catalase-peroxidase) is examined, and for detecting low-level INH resistance, the promoter region of the *inhA* gene (coding for the NADH enoyl ACP reductase) is evaluated [62].

GenoType MTBDR*sl* version 1 was the first commercial nucleic acid-based test for the detection of second-line drug resistance and was recommended by the WHO as the reference test for XDR-TB until it was replaced by the improved second version of GenoType MTBDR*sl*, enabling the detection of mutations involved in resistance to injectable drugs and FQs [63]. The assay is flexible, choosing between manual or automated procedures based on the sample throughput needs of the setting. The test results are generally available within a few hours (~five hours) compared to conventional methods that take several weeks [63]. Furthermore, although MTBDR*sl* has low diagnostic sensitivity for detecting ethambutol and aminoglycoside resistance [64], it remains a crucial reference tool for rapid detection of pre-XDR-TB from smear-positive clinical samples, given that alternative tests have a prolonged turnaround time in the laboratory and are technically challenging to perform. Negative results, however, require further investigation, as resistance to second-line drugs may still be present but not detected by the test. Smear-negative sputum samples need to be cultured before MTBDR*sl* testing, producing an additional limitation to the application of this assay [65]. The overall sensitivities of the GenoType MTBDR tests vary from 96% for RIF to 44% for kanamycin (KAN), while specificities range from 99% for INH, RIF, AMK, and KAN to 79% for EMB [62,64,66]. The assay has historically complemented frontline diagnostic assays such as Xpert MTB/RIF by confirming RIF resistance and providing additional information on INH susceptibility and pharmacogenetic information that may be essential for guiding appropriate treatment [67].

### 10.2. Genoscholar Assays (NIPRO Corporation, Osaka, Japan)

Genoscholar assays are probe-based drug susceptibility technologies that employ reverse hybridisation techniques in which amplicons are hybridised on a nitrocellulose membrane strip. The assay results are then interpreted based on the presence or absence of bands at various points on the strip, enabling the simultaneous detection of MTB and mutations associated with resistance to anti-TB drugs such as RIF, INH, PZA, FQ, and KAM [68]. The Genoscholar line probe assays include Genoscholar NTM+MDRTB II, Genoscholar PZA TB II, and Genoscholar FQ+KM-TB II.

Genoscholar NTM+MDRTB II is a first-line line probe assay that identifies not only MTBc but also *M. avium*, *M. intracellulare* and *M. kansasii*, while detecting RIF and INH resistance [9]. Introduced in 2011 by the Nipro Corporation, the Genoscholar NTM+MDRTB assay is designed as a two-step process with two separate amplification reactions. The first step is the detection of *Mycobacterium* species; the second step is the detection of drug resistance [69,70,71]. The assay’s performance in identifying *Mycobacterium* species in clinical isolates was comparable to conventional identification methods. Furthermore, compared with standard DST, the assay showed a sensitivity of 98.9% and specificity of 97.3% for detecting RIF resistance and a sensitivity and specificity of 90.6% and 100%, respectively, for INH resistance [71].

Genoscholar PZA TB II is a relatively new rapid molecular drug susceptibility assay that detects mutations associated with resistance to PZA. It targets a 700-base-pair fragment covering the entire *pncA* gene and promoter region up to nucleotide–18 of the wild-type H37Rv reference strain, which is known to harbour resistance-associated mutations [72]. Direct and indirect specimens are suitable for use with Genoscholar assays, with test results available within a day. However, Genoscholar PZA TB II performance data on direct specimens are limited, and the recommendation currently only advocates using MTB isolates to detect PZA resistance; thus, this test is appropriate for use only where culture facilities are available. The overall sensitivity and specificity of Genoscholar PZA TB II for detecting PZA resistance are 81% and 98%, respectively [9].

Genoscholar FQ+KM-TB II is a second-line line probe assay that detects resistance to fluoroquinolone (FQ) and kanamycin (KAM) [73], with FQ resistance detection performance of 93% sensitivity and 100% specificity, and a turnaround time of 6 h [74]. The assay can be performed using raw sputum samples as well as culture isolates. The Genoscholar FQ+KM-TB II is still under evaluation for its clinical performance regarding kanamycin resistance detection [75,76].

Although line probe assays (LPAs) are more complex compared to Xpert MTB/RIF, they can detect resistance to a wider range of first- and second-line agents, such as RIF, INH, FQ, and injectable agents, and the test platforms are designed for reference laboratories in countries with a high TB burden. The use of second-line LPAs in routine care could improve the time to diagnosis of resistance to second-line FQ and injectable agents.

### 10.3. GeneXpert Assays (Cepheid Inc., Sunnyvale, CA, USA)

Xpert MTB/RIF assay is an automated cartridge-based assay that employs real-time polymerase chain reaction (PCR) on the GeneXpert platform to identify MTB and detect mutations associated with RIF resistance directly from respiratory samples (e.g., sputum). The sensitivity and specificity for detecting RIF resistance were 96% and 95%, respectively, compared to culture as the reference standard. Xpert MTB/RIF is an ideal first-line diagnostic tool for pulmonary TB cases because of its excellent sensitivity and specificity for diagnosing pulmonary TB, and ability to simultaneously detect RIF resistance in less than 2 h [77,78,79]. Xpert MTB/RIF is widely used in both high- and low-TB burden regions and has greatly improved the diagnosis of TB and RIF resistance detection rate globally. Although designed for point of- care (POC), the Xpert MTB/RIF test is mainly used in centralised laboratories [80]. Moreover, Xpert MTB/RIF does not detect INH resistance, despite this form of resistance being present in 8% of TB cases worldwide [81]. Nevertheless, to circumvent some limitations observed with Xpert MTB/RIF, such as the reported false-positive Xpert MTB/RIF results caused by the silent mutations (e.g., at codon 514 of the *rpoB* gene) [82], the false-negative results due to failure to detect mutations conferring resistance to RIF outside the *rpoB* hotspot region [83], and the inability to detect INH resistance, Xpert MTB/RIF Ultra and MTB/XDR assays were introduced [84].

Xpert MTB/RIF Ultra assay, also called Xpert Ultra, uses the same GeneXpert platform as the Xpert MTB/RIF test and was developed to improve the sensitivity and reliability of detecting MTBc and RIF resistance simultaneously. To improve sensitivity, Xpert Ultra uses two multicopy amplification targets (IS6110 and IS1081) and a larger PCR chamber; thus, Xpert Ultra has a lower limit of detection than Xpert MTB/RIF (16 and 131 CFU/mL, respectively). Another improvement in the Xpert Ultra is that the analysis is based on melting temperature, which allows for better differentiation of resistance-conferring mutations. Xpert Ultra has better sensitivity (88%) than Xpert RIF (72%) and culture (44%). However, the specificity remained lower (98%) than that of the Xpert (100%) and culture (100%) [85]. Xpert MTB/RIF Ultra has the potential to reduce the number of missed negative smears, therefore lowering patient care costs through the effective detection of TB and RIF resistance in a single test.

Xpert MTB/XDR assay: Xpert MTB/XDR sets new standards by detecting mutations associated with resistance towards INH (*inhA promoter*, *katG*, *fabG1*, *oxyR-aphC intergenic region*), FQs (*gyrA* and *gyrB*), second-line injectable drugs (amikacin, kanamycin, capreomycin) (*rrs* and *eis promoter*) and ethionamide (ETH) (*inhA promoter*) in a single assay, while, Xpert MTB/RIF and Xpert MTB/RIF Ultra only diagnose TB and detect resistance to RIF without providing information regarding resistance to other anti-TB drugs [86]. In contrast, information on each class of anti-TB drug is essential for initiating efficient and adequate treatment, breaking the transmission chain, and reducing severe illness and related death. The sensitivity and specificity of Xpert MTB/XDR, irrespective of rifampicin resistance, were 94% and 98%, respectively, against culture-based pDST. Whereas for FQs resistance, Xpert MTB/XDR sensitivity and specificity were 93% and 98% compared to pDST [87]. In people with known rifampicin resistance, Xpert MTB/XDR sensitivity and specificity for ETH resistance were 98% and 99.7% respectively. For amikacin resistance, the Xpert MTB/XDR sensitivity and specificity were 86% and 99% [88]. This assay is recommended as a reflex text in people diagnosed with rifampicin-resistant tuberculosis in countries with a high burden of DR-TB. The most significant advantage of Xpert MTB/XDR is its suitability for point of care (POC) implementation based on the assay’s capability to profile DST for first and second-line anti-TB drugs without the need for complex laboratory infrastructure [86,89]. Xpert MTB/XDR provides a faster time to result for clinically actionable DST, an easy-to-use process, and is performed on existing GeneXpert platforms. Innovative research is still required to expand resistance calls to include the novel and repurposed anti-TB agents, and to establish the most suitable placement of this test within diagnostic algorithms and laboratory workflows. As a direct reflex test to Xpert MTB/RIF Ultra, the XDR assay has the potential to link patients to early and appropriate treatment [86].

### 10.4. Abbott RealTime MTB and MTB RIF/INH (Abbott Molecular, Des Plaines, IL, USA)

Abbott RealTime MTB RIF/INH is an automated and moderate complexity nucleic acid amplification test (NAAT) that detects not only MTB and RIF resistance but also INH resistance. It is a qualitative detection assay targeting two different MTB-specific DNA sequences (IS6110 genetic element and *pab* gene) from direct or indirect specimens [90,91]. The test uses eight dye-labelled probes to detect mutations in the RIF-resistance determining region (RRDR) of the *rpoB* gene for RIF resistance and four probes for INH resistance detection, with two probes each for the *katG* and *inhA* genes [92]. Studies reported that Abbott RealTime MTB RIF/INH assays have high sensitivity and specificity in MTB diagnosis and provided reliable INH and RIF resistance detection, having a similar diagnostic performance as the Xpert MTB/RIF assay with the additional advantages of a higher throughput and INH resistance detection [90]. Abbott RealTime MTB is performed using the m2000™ system analyser. This system can perform multiple assays, including Abbott RealTime HIV testing, as well as capabilities for the detection of hepatitis C and B, cytomegalovirus; Epstein-Barr virus; human papillomavirus (HPV); Chlamydia trachomatis, *Neisseria gonorrhoeae*, and severe acute respiratory syndrome coronavirus 2 (SARSCoV-2). This cross-purpose solutioning may be of particular importance in settings with limited space, a high burden of infectious diseases, and the need to provide a service across disease programmes.

The RealTime MTB RIF/INH resistance assay is a sensitive, robust, and reliable test for real-time simultaneous detection of first-line anti-TB RIF and INH in clinical samples. The sensitivity and specificity of Abbott RealTime for detecting TB were 96% and 97%, respectively, and based on assay performance metadata analysis of Abbott RealTime MTB RIF/INH, compared to reference pDST, the pooled sensitivity and specificity for RIF resistance detection were 94% and 100%, respectively, and for INH resistance detection, the sensitivity and specificity were 89% with 99%, respectively [93,94]. The turnaround time from sample acquisition to results generation is approximately 10.5 h, with a detection threshold of 17 CFU/mL for MTBc and 60 CFU/mL for RIF/INH resistance detection [9,95]. Abbott m2000 systems are high-throughput laboratory instruments that require significant infrastructure and must be installed in laboratories that can accommodate a molecular workflow, including separate, dedicated preparation and amplification compartments.

### 10.5. FluoroType MTBDR (RIF, INH) (Bruker/Hain Lifescience, Nehren, Germany)

Bruker-Hain Diagnostics offers two automated real-time nucleic acid amplification assays of moderate complexity; the FluoroType MTB for the detection of MTBc and the FluoroType MTBDR for the detection of resistance to RIF and INH. FluoroType MTB targets the IS6110 DNA insert for the detection of MTBc, while FluoroType MTBDR targets the *rpoB* gene for the detection of MTBc and RIF resistance, as well as the *inhA* promoter and *katG* gene for the detection of INH resistance [96]. FluoroType MTB coupled with MTBDR is a rapid, closed-tube, centralised molecular assay with automated DNA extraction PCR set-up method (GenoXtract 96 instrument [GXT96]) and automated result interpretation platforms, thereby removing operator interpretation partiality. This high throughput assay can assess a large number of samples per run (96 samples including controls) with time to result within 4 h [97]. The FluoroType assay differentiates between high- and low-level INH resistance and also reports the specific mutations identified. The diagnostic accuracy of FluoroType MTB for detecting MTBc in smear-positive specimens was excellent, with sensitivity and specificity of 92.1% and 98.9%, respectively [94]. Compared to pDST, the sensitivity and specificity of FluoroType MTBDR in detecting RIF resistance from direct specimens were 97% and 100%, respectively [98], and from culture-isolated specimens, the sensitivity and specificity were 99% and 100%, respectively [96]. For INH resistance detection, the sensitivities of the assay using direct and indirect specimens were 70% and 92%, respectively, and specificity was 100% in both cases [94,96,98]. WHO recommended FluoroType MTBDR as a test for pulmonary TB diagnosis and RIF and INH resistance detection in people with signs and symptoms of pulmonary TB; however, the use in people with extrapulmonary TB and testing on non-sputum samples needs further investigation.

### 10.6. BD MAX Multi-Drug Resistant Tuberculosis (Max MDR-TB) Assay (Becton Dickinson)

Max MDR-TB is an automated qualitative diagnostic test for detection of MTBc DNA in raw sputum or processed samples [99]. This test meets the WHO’s consolidated guidelines for TB diagnosis by enabling rapid detection of TB using the multicopy genomic elements *IS6110* and *IS1081*, together or separately [9]. To detect resistance to RIF, the test targets codons *507-533* of the *rpoB* gene, and to detect resistance to INH, the test targets both the *inhA* promoter region and codon *315* of the *katG* gene (79). The reported LoD to detect MTB is 0.5 CFU/mL, and 6 CFU/mL for RIF and INH detection. BD MAX MDR-TB test performs well for the diagnosis of TB and DR-TB compared with culture and pDST, as proven by a recent study that evaluated the diagnostic accuracy of the assay revealing a sensitivity of 90.6% and specificity of 98.5% for the detection of MTB in pulmonary specimens and a sensitivity of 82.5% and specificity of 98.9% for extrapulmonary specimens [100]. For RIF resistance, the sensitivity and specificity compared to pDST were 90% and 95%, respectively, while for INH resistance, the sensitivity and specificity were 82% and 100%, respectively [94]. Similar reports on the diagnostic accuracy of BD MAX MDR-TB assay have been reported in a variety of geographical settings and TB prevalence [101,102]. The BD MAX platform is integrated with a DNA extraction system and can process up to 24 samples at a time and discriminate and report high- (*katG*) and low-level (*inhA*) INH resistance with a turnaround time of four hours. BD MAX MDR-TB is a reliable, rapid, user-friendly assay for detecting MTBc in extrapulmonary and pulmonary specimens and profiling RIF and INH resistance. It can be used as an alternative to the Xpert system assays, as is being implemented in South Africa.

### 10.7. Cobas MTB and MTB RIF/INH (Roche Molecular Diagnostics, Pleasanton, CA, USA)

Performed using Cobas 5800/6800/8800 platforms, Cobas MTB-RIF/INH is an automated, qualitative real-time PCR assay designed as a reflex test together with Cobas MTB to detect eighteen mutations associated with RIF resistance (*rpoB* gene) and seven mutations associated with INH resistance (*katG* gene and promoter region of *inhA* gene) using multiple primers and mutation-specific probes [103]. Assays are performed on AFB positive or negative smears, raw sputum, digested and decontaminated sputum (treated with N-acetyl-L-cysteine/NaOH), and bronchoalveolar lavage (BAL) samples. The reported detection limit for MTB ranges from 7 to 8 CFU/mL, for RIF resistance from 94 to 182 CFU/mL, and for INH from 12 to 27 CFU/mL, depending on the sample type [103]. The clinical performance of Cobas MTB RIF/INH has been estimated at 97.2% sensitivity, 98.6% specificity for RIF resistance, 96.9% sensitivity, and 99.4% specificity for INH resistance detection [104]. The Cobas MTB-RIF/INH test can also identify and differentiate between RIF and INH monoresistant tuberculosis and MDR-TB in the majority of samples studied, enabling appropriate therapeutic interventions based on current guidelines and recommendations.

### 10.8. Loop-Mediated Isothermal Amplification (LAMP)

Loop-mediated isothermal amplification (LAMP) is a manual nucleic acid-based method used to identify mycobacteria in less than an hour by reading under ultraviolet light. TB-LAMP has the advantage of being relatively high performance, not requiring sophisticated instrumentation and infrastructure, and similar to those of sputum smear microscopy [105]. The TB-LAMP method was endorsed by the WHO in 2016 for the diagnosis of pulmonary TB in adults, and has been a primary diagnostic test for pulmonary tuberculosis in many low-resource settings [106]. Loop-mediated isothermal amplification was thereafter evaluated for the diagnosis of MDR-TB and demonstrated high sensitivity and specificity for detecting resistance to RIF and INH. The sensitivity and specificity of MDR-LAMP were 93.1% and 92.3% for the detection of INH resistance (*katG* and *inhA* gene mutations), respectively, and 89.1% and 88.9% for detecting *rpoB* gene mutation, respectively, when compared to whole genome sequencing as the reference standard [107]. MDR-LAMP is an accessible DST method for the identification of RIF/INH resistance, which does not require specialised equipment and can meet the diagnostic demand in resource-limited areas where DR-TB is endemic, such as LMICs.

## 11. Chip-Based Real-Time Micro PCR Assays

### Truenat MTB-RIF-Dx (Molbio Diagnostics, Goa, India)

The Truenat MTB and MTB Plus assays use chip-based real-time micro polymerase chain reaction (PCR) assays for the semiquantitative detection of MTBc in direct sputum samples, and results can be obtained in less than an hour. They are automated diagnostic tools with battery-operated devices for extraction, amplification, and detection of specific genomic DNA loci [108]. The assays are designed to be processed in peripheral laboratories with minimal infrastructure and minimally trained technicians; however, the technique requires pipetting skills. The Truenat MTB-RIF-Dx assay performed similarly to MTBDRplus for RIF resistance mutation detection and was comparable to that of the Xpert MTB/RIF assay [109]. Truenat MTB-RIF-Dx does not detect the *rpoB Q432K* and *S441L* mutations [110]. However, these mutations are rare, with less than 0.7% frequencies globally [110,111,112].

The introduction of molecular-based DST TB has revolutionised TB diagnosis, with significant improvement in the detection of resistance to RIF in a shorter time. It also heralded a substantial improvement in treatment outcomes due to the more rapid initiation of appropriate treatment. However, these rapid molecular DST methods have limitations in guiding therapeutic decisions in the context of widely implemented novel, short, all-oral, bedaquiline-containing regimens such as BPaL/M. There is presently no rapid molecular test that can simultaneously detect resistance to all current first- and second-line TB drugs, nor to the newly proposed anti-TB drugs (e.g., bedaquiline, pretomanid, linezolid, delamanid). The assays have limited capacity in terms of the number of anti-TB drugs and genetic regions to analyse [113]. Therefore, innovation toward more powerful technologies is required, and to address this, next-generation sequencing (NGS) tools are quite promising in covering a large number of anti-TB drugs for rapidly generating comprehensive resistance profiles.

## 12. Sequence-Based Assays

### Deeplex MYC-TB Assay (Genoscreen, France)

The Deeplex Myc-TB assay uses next-generation targeted deep sequencing for simultaneous prediction of resistance to up to 15 anti-TB drugs, MTBc genotyping, and differential identification of mycobacteria. The assay is performed using clinical samples and demonstrates excellent diagnostic performance with an average sensitivity of 95% and specificity of 97% compared to pDST [114]. The Deeplex-MycTB assay can be performed directly on clinical specimens and has the potential to rapidly detect drug-susceptible TB, MDR-TB, and XDR-TB, with more comprehensive coverage of 18 resistance targets, and enables the detection of bacterial sub-populations [115]. Detection of different sub-populations/heteroresistance is important as its presence complicates treatment and threatens successful outcomes [3]. Heteroresistance has been linked with persistent infection and an increased risk of treatment failure [116]. Hence, it is important that it is detected early, allowing for a differentiated treatment approach for improving treatment outcomes. This is an important advantage over other molecular DST assays in pursuit of highly personalised therapy. In addition to comprehensive interrogation of genomic regions, this assays compares the analysed regions with integrated reference database sequences [117], providing data on the confidence of the mutations detected. Further, as the assay covers larger regions of genes, i.e., *rpoB*, *katG*, *embB*, *rrs*, it has the ability to detect non-canonical/uncharacterised mutations [117]. Non-canonical mutations can still be of clinical relevance, thus their detection is important for guiding further evaluating by pDST and informing therapeutic decisions in near real time. Foremost, the assay significantly reduces the turnaround time for clinically actionable DST results and may substantially reduce the load of pDST within DR-TB programmes.

## 13. Next-Generation Sequencing (NGS)

Next-generation sequencing (NGS) is a new approach to detecting key mutations associated with MTB drug resistance. NGS and its associated technologies have demonstrated exceptional potential for reliable and comprehensive prediction of resistance in MTB strains, enabling accurate clinical decisions [118]. A recent study showed that NGS technology was highly sensitive in detecting mutations associated with resistance to INH, RIF, ofloxacin, levofloxacin, and moxifloxacin (100%), as well as streptomycin (96.7%). Sensitivity to ethambutol and kanamycin was lower (87.5% and 88.9%). Sensitivity to amikacin and capreomycin was similar (60.0%), and the lowest sensitivity was observed with PZA (29.4%), while specificity was over 90% for all anti-tuberculosis drugs [119].

NGS technology, specifically whole genome sequencing (WGS), offers the most comprehensive approach to molecular-based DST for TB. It allows examination of all mutations that could potentially confer drug resistance to TB, thereby enabling treatment individualisation. WGS helps in the tracking of heteroresistance, transmission patterns, mixed infections, disease outbreaks, and surveillance and provides additional clinical advantages [120,121]. In developed countries, WGS is used as the reference method for DR-TB diagnosis and to inform treatment regimens [122,123]. On the other hand, in LMICs and in regions where TB is widespread and where it is most needed, WGS technologies are less widely used partly due to the necessity for specialised facilities, qualified personnel, and bioinformatic data analysis capabilities and relatively high cost. WGS is a robust and efficient tool to guide DR-TB treatment; however, this technology has limitations, such as high rates of failed sequencing reads when insufficient genomic material is present in direct clinical specimens. Investigations are directed towards optimising methods such as the use of enzymatic lysing, bead beating, heat, and enrichment of drug-resistant genes to overcome some of the challenges.

## 14. Mass Spectrometry-Based DST

Mass spectrometry is an analytical technique in which samples are ionised into charged molecules and the ratio of their mass-to-charge (*m*/*z*) is measured. Matrix assisted laser desorption ionisation time of flight mass spectrometry (MALDI-TOF-MS) is used to detect the probable proteins or oligonucleotides related to the resistance based on the specific peptide mass fingerprinting in the protein database, and the drug susceptibility of MTB can be resolved. MALDI-TOF-MS directly detects the molecular weight of the molecules in biological samples, which can be used for single-nucleotide polymorphism, methylation, and microbial detection and typing [124]. Using clinical sputum specimens, MALDI-TOF-MS showed better sensitivity and specificity than the COBAS TaqMan MTB test. Thus, the MALDI-TOF-based MTB assay is a promising new strategy for the rapid, simultaneous identification of MTB and drug resistance from sputum within laboratories with MS capabilities [125].

## 15. Comparison of Phenotypic, Genotypic-, and Sequencing-Based Dst Assays

Recent studies demonstrated the agreement between Xpert, line probe assays (LPAs), and WGS compared to pDST for predicting resistance DR-TB. The average agreement in the number of drugs prescribed in genotypic regimens ranged from just 49% for Xpert and 63% for LPAs to 93% for WGS. Only the WGS regimens did not contain any drugs to which pDST showed resistance [126]. Targeted NGS analysis reached a detection limit of at least 1% mutant variants, equalling the 1% critical concentration that applies to pDST [73]. However, pDST is still useful for identifying resistance generated by novel mutations and to detect resistance caused by known resistance mutations that occur at frequencies below the detection limit of sequencing. Furthermore, pDST is needed for drugs for which a molecular method is not available yet and remains crucial for determining susceptibility to new and repurposed classes of anti-TB drugs. Therefore, genotypic and phenotypic DST are best viewed as complementary in the diagnosis and treatment of DR-TB [27].

## 16. Conclusions

Effective TB control relies on our ability to rapidly detect TB and establish susceptibility to commonly used drugs. With an expanding diagnostic armamentarium, there has been substantial progress towards rapid, reliable, accurate, and actionable diagnostic strategies. While this review focused primarily on WHO-endorsed diagnostic tools, we included coverage of promising novel technologies including phage assays and mass spectrometry-based DST. Meta-analyses of the diagnostic performance of each of the reviewed assays, especially from real-world data, will strengthen our understanding of the implementation potential of available diagnostic solutions. Molecular diagnostic tests, especially those of low complexity, have allowed for the decentralisation of TB diagnostic services, and in some instances promise to deliver point of care diagnostic solutions for TB detection and resistance profiling. However, the shifting treatment landscape, and the global implementation of regimens comprising novel drugs, has generated new diagnostic challenges, for which molecular diagnostic solutions do not yet exist. For key drugs, such as bedaquiline, pretomanid, and delamanid, the return to culture-based phenotypic DST is a reminder of the progress that is still needed to materialise the goal of rapid phenotypic drug resistance profiling, and of the limitations of our reliance on molecular diagnostic strategies alone. Indeed, both genotype and phenotype offer unique and important information to guide treatment and enable vigilance for emergent resistance. Diagnostic strategies for DR-TB will therefore need to be tailored to local disease epidemiology, laboratory infrastructure, availability of diagnostic solutions and technical support, changes in therapeutic strategies, and the challenges presented by HIV/TB co-infection, multimorbidity, and the rise of antimicrobial resistance more broadly. These represent key priorities for future discovery and implementation research.

## Figures and Tables

**Table 1:** Performance of WHO-endorsed genotypic and phenotypic diagnostics for drug-resistant TB diagnosis and drug-susceptibility testing.

Assay (Manufacture, Year)	Test Type	Gene Target (Anti-TB Drug)	(% Sensitivity, Specificity)	Strengths	Limitations
Probe-based genotypic Assays					
Xpert MTB/RIF (Cepheid, Sunnyvale, CA, USA, 2010)	Cartridge-based real time PCR	*rpoB* (RIF)	MTBC: (85, 98) RIF resistance: (96, 98)	One-step process, automated Can be performed directly from concentrated sputum sediment Rapid turnaround time, <2 h Can be performed in a BSL2 facility High sensitivity and specificity Multi-platform: can be used for HIV, hepatitis C diagnosis and viral load monitoring Can be used on extrapulmonary TB samples Little hands-on time Point-of-care capability FDA cleared; widely available in clinical and public health laboratories	Expensive Relies on stable power supply Cannot be used to monitor treatment response Requires annual calibration Limited to canonical mutation detection Difficult to detect heteroresistance Does not test for new and repurposed drugs
Xpert MTB/RIF Ultra (Cepheid, 2017)	Cartridge-based real time PCR and melt curve	*rpoB* (RIF)	MTBC: (90, 96) RIF resistance (94, 98)		
Xpert MTB/XDR (Cepheid, 2021)	Cartridge-based real time PCR	*inhA*, *fabG1*, *oxyR-ahpC*, *katG* (INH, ETH *), *gyrA*, *gyrB* (FQs), *rrs*, *eis* (SLIDs)	MTBC: (100, 100) Drug resistance: INH (94.2, 98); FQs (93.1, 98.3); AMK (86.1, 98.9); ETH (98, 99.7); KAN (98.1, 97), CAP (70, 99.7)		
BD MAX MDR-TB (Becton Dickinson, North Charleston, SC, USA, 2021)	Automated multiplexed real-time PCR on high throughput platform	*rpoB* (RIF), *inhA*, *katG* (codon 315) (INH)	MTBC: (90.6, 98.5) Drug resistance: RIF (90, 95), INH (82, 100)	Can be performed directly from the raw sputum Can be performed on decontaminated sputum for FluoroType MTBDR Can be performed on bronchoalveolar lavage fluid High sample throughput Each assay has specific multi-platform benefits for HIV-1, HBV, HCV, HPV, SARS-CoV2	Relies on stable power supply Expensive equipment Specialised training is required Limited to canonical INH and RIF resistance mutation detection
Cobas MTB-RIF/INH (Roche Diagnostics, Basel, Switzerland, 2021)	Automated NAAT on high throughput platform	*rpoB* (RIF), *inhA*, *katG* (INH)	MTBC: (89.2, 98.6) Drug resistance: RIF (97.2, 98.6), INH (96.9, 99.4)		
RealTime MTB RIF (Abbott Molecular, Des Plaines, IL, USA, 2019)	Automated NAAT on high throughput platform	*rpoB* (RIF), *inhA*, *katG* (INH)	MTBC: (92.4, 95.4) Drug resistance: RIF (94.8, 100), INH (88.3, 94.3)		
FluoroType MTBDR (Hain LifeScience, Nehren, Germany, 2021)	Automated NAAT on high throughput platform	*rpoB* (RIF), *inhA*, *katG* (INH)	MTBC: (96.1, 100) Drug resistance: RIF (98.9, 100), INH (91.7, 100)		
TRUENAT MTB- RIF Dx (Molbio Diagnostics, Goa, India, 2020)	Chip-based real-time micro-PCR Battery operated	*rpoB* (RIF)	MTBC: (100, 100) RIF resistance (84, 97);	Battery operated Rapid turnaround time, <1 h Can be performed on raw sputum and other extrapulmonary body fluids Multi-platform: can test for hepatitis C, HPV, SARS-CoV2	Labour-intensive and requires skilled personnel
GenoType MTBDR*plus* (Hain Lifescience, 2008)	Line probe assay based on reverse-hybridisation DNA strip technology	*rpoB* (RIF), *inhA*, *katG* (INH, ETH *)	MTBC: (82.7, 98.9) Drug resistance: RIF (98.2, 97.8); INH (95.4, 98.8)	Can be performed on pulmonary specimen or culture isolate Results are obtained in 5 h Can detect heteroresistance in INH and RIF	Limited to specific canonical mutations Not as fast as Xpert Does not test for new and repurposed drugs Requires skilled personnel Requires BSL3 facility Requires expensive equipment and complex laboratory infrastructure Frequent uninterpretable/indeterminate results
GenoType MTBDR*sl* v 2.0 (Hain Lifescience, 2016)	Line probe assay based on reverse-hybridisation DNA strip technology	*gyrA*, *gryB* (FQs), rrs, eis (SLIDs)	Drug resistance: FQ (100, 98.9); AMK (93.8, 98.5); CAP (86.2, 95.9)		
Genoscholar NTM+MDRTB II (Nipro, Osaka, Japan, 2021)	Line probe assay based on reverse-hybridisation DNA strip technology	*rpoB* (RIF), *katG*, *inhA* (INH), *pncA* (PZA)	DR-TB, RIF: (96.5, 97.5), INH: (94.9, 97.6)	NTM+MDRTB II also identifies *M. avium*, *M. intracellulare*, *M. kansasii*	Target coverage is limited to specific mutations. Difficult to detect heteroresistance
Genoscholar PZA TB II (Nipro, 2021)	Manual reverse hybridisation assay	*pncA* (PZA)	PZA: (81%, 98%,)	High complexity reverse hybridisation nucleic acid amplification test	PZA-TB assay recommended only on culture isolates
FQ+KM-TB II (Nipro, 2021)	Reverse hybridisation assay	*gyrA*, *gyrB*, (FQs) *eis*, *rrs* (SLIDs)	FQ: (93.0, 100) SLIDs (NA)	Provides rapid FQ resistant detection, essential for initial selection of the most appropriate DR-TB regimen	Not as fast as Xpert Does not test for new and repurposed drugs
Sequencing-based DST Assays					
Deeplex Myc-TB (Genoscreen, Lille, France, 2023)	Targeted NGS	*rpoB* (RIF), *inhA*, *fabG1*, *ahpC*, *katG* (INH, ETH^*^), *pncA* (PZA), *embB* (EMB), *gidB*, *rpsL* (STR), *gyrA*, *gyrB* (FQs), *rrs* (AMK, STR, KAN, CAP), *eis* (KAN), *tlyA* (CAP), *ethA*, (ETH), *rrl*, *rplC* (LZD), *rv0678* (BDQ, CFZ))	RIF (99.4, 98.8); INH (98.3, 98.4); PZA (85.7, 100); EMB (92.2, 90.7); STR (90.7, 98.9) FQs (91.7, 99.2); AMK (100, 100); KAN (88.9, 100); CAP (93.8, 97.4); ETH (92.6, 68) LZD (NA, 100)	High coverage depths for detection of high-confidence mutations and minor variants Resistance detection to new and repurposed drugs Detection of other mycobacterial species Lineage identification Detection of heteroresistance Identification of non-canonical mutations Spoligotyping	Poor performance in sputum with low bacillary load Expensive kit Limited gene regions assessed Throughput of 45 samples may be too many in low in TB burden settings and too few in TB endemic settings
Ion AmpliSeq (Thermo Fisher Scientific, Waltham, MA, USA)	Targeted NGS	*embB* (EMB), *eis* (AMK, KAN, CAP), *gyrA* (FQs), *inhA*, *katG* (INH), *pncA* (PZA), *rpoB* (RIF), *rpsL* (STR)	RIF (100, 100); NH (100, 100) EMB (92.9, 93.8); PZA (100, FQs (100, 100); AMK (100, 100); CAP (66.7, 100); KAN (66.7, 96.7); STR (92.9, 100)	Small sample input Scalable and customisable panels Fast, automated workflow	Has short read length (~ 400 nucleotides per read) compared to other sequencing methods
Phenotypic DST Assays					
Löwenstein-Jensen (LJ)	Solid culture	-	-	Capable of testing all anti-drugs with established CC and CB Can be used for MIC determination	Results take up to 6 weeks Requires cultured isolate from solid media LJ is currently not standardised to test for new and repurposed drugs
Middlebrook 7H10, 7H11	Solid culture	-	-	Capable of testing all anti-drugs with established CC and CB including new and repurposed drugs except clofazimine Can be used for MIC determination	
MGIT 460/960 (Becton Dickinson, 2007)	Liquid media	-	MTBC: (95.2, 99.2)	Rapid turnaround time of 4-24 days for DST and MIC Can test for new and repurpose drugs High reproducibility	Easily contaminated Requires a positive culture isolate before DST and MIC test Labour intensive Requires well-trained personnel Requires BSL3 facility Requires complex laboratory infrastructure
MODS	Liquid culture and light microscopy	-	DR-TB Pooled (94.4, 91.8)	Can be performed using decontaminated and concentrated sputum sediment First and second-line DST, including BDQ, LZD, and DLM Rapid turnaround time	High risk of contamination Requires additional skills in using inverted microscopy Additional consumables (microtiter plates) that may be difficult to obtain Requires daily examinations after day 5 of incubation Time-consuming

Abbreviations: AMK, amikacin; BDQ, bedaquiline; BSL2/3, Biosafety level 2/3; CAP, capreomycin; CFZ, clofazimine; DLM, delamanid; DR-TB, drug resistant TB; DST, Drug Susceptibility Testing; DNA, Deoxy-ribonucleic acid; EMB, ethambutol; ETH, ethionamide; FQs, fluoroquinolones; HIV, human immunodeficiency virus; HPV, human papilloma virus; INH, isoniazid; KAN, kanamycin; LZD, linezolid; LFX, levofloxacin; MDR-TB, Multi Drug-Resistant TB; MTBC, *Mycobacterium tuberculosis* complex; MXF, moxifloxacin; STR, streptomycin; PCR, polymerase chain reaction; PZA, pyrazinamide; RIF, rifampicin; SLIDs, second-line injectable drugs. * ETH has cross-resistance through *inhA* and *fabG1.*

## Data Availability

Not applicable.
